# Supplementary material for: Plant-insect interactions patterns in three European paleoforests of the late-Neogene—early-Quaternary
Source: PeerJ. 2018 Jun 20;6:e5075. doi: 10.7717/peerj.5075 (PMC6015487; doi:10.7717/peerj.5075)
Supplement: Supplemental Information 1 — These are based on the following methods: co-existing approach (CoA), leaf margin analysis (LMA), Climate Leaf Analysis Multivariate Program (CLAMP), European Leaf Physiognomic Approach (ELPA), Climatic Amplitude Method (CAM). For further details, please note that data for Willershausen and Berga come from Thiel et al. (2012) and data for Bernasso come from a- Leroy & Roiron (1996), b- Girard et al. (submitted). [file peerj-06-5075-s001.docx]

| **Climatic parameter** (method) | Estimate for **Willershausen** | Estimate for **Berga** | Estimate for **Bernasso** |
| --- | --- | --- | --- |
| Mean annual temperature (CoA) | 13.6 – 15.6 | 13.6 – 16.6 | 14.8 – 17.8^a,b^ |
| Mean annual temperature (LMA) | 9.3 – 11.9 | 6.2 – 11.4 | 6.66 – 11.39^b^ |
| Mean annual temperature (CLAMP) | 10 – 12.2 | 7.7 – 10.1 | 3.79 – 8.58^b^ |
| Mean annual temperature (ELPA) | 9.7 – 11.9 | 6.3 – 8.5 | n.a. |
| Mean annual temperature (CAM) | n.a. | n.a. | 11 – 20^b^ |
| Mean temperature of the coldest month (CoA) | 0.6 – 1.7 | 0.6 – 1.7 | n.a. |
| Mean temperature of the coldest month (CLAMP) | 1.3 – 5.1 | -1.7 – 2.1 | -8.23 – -1.89^b^ |
| Mean temperature of the coldest month (ELPA) | -0.5 – 3.7 | -6.4 – -2.2 | n.a. |
| Mean temperature of the coldest month (CAM) | n.a. | n.a. | -2 – 5^b^ |
| Mean annual precipitation (CoA) | 897 – 1151 | 897 –1297 | 1020 – 1220^a^ |
| Mean annual precipitation (CLAMP) | n.a. | n.a. | 1626 – 1752.9^b^ |
| Mean annual precipitation (CAM) | n.a. | n.a. | 700 – 1350^b^ |
