# Supplementary material for: Plant-insect interactions patterns in three European paleoforests of the late-Neogene—early-Quaternary
Source: PeerJ. 2018 Jun 20;6:e5075. doi: 10.7717/peerj.5075 (PMC6015487; doi:10.7717/peerj.5075)
Supplement: Supplemental Information 2 — ¶ not found in the collections; * new ichnospecies type; § named after recent species with the addendum fossilis; H: holotype; P: paratype. [file peerj-06-5075-s002.docx]

Table S2: Plant-Insect interaction and herbivory pattern observerd and originally described by Adolf Straus (1977) in his first comprehensive work on leaf interactions from Willershausen.

^¶^ not found in the collections; ^*^ new ichnospecies type; ^§^ named after recent species with the addendum *fossilis*; H holotype; P paratype;

| **Coll. No.** | **Ichnotaxon** | **Type** | **Straus orginal Ref.** | **Plate Ref.** | **DT** | **FFG** | **Host plant** | **Culprit** | **Comments** |
| --- | --- | --- | --- | --- | --- | --- | --- | --- | --- |
| 17381^§^ | cf. *Taphrina* sp. |  | Straus (1977; Taf. 1, Fig. 19) |  | 16 | Galling | *Parrotia* sp. | Ascomycetes: Exoascaceae |  |
| 18419^§^ | cf. *Taphrina* sp. |  |  |  | 2 | Fungal | *Populus* sp. | Ascomycetes: Exoascaceae |  |
| Fu 174 | *Gymnosporangium* sp. |  |  |  | 80 | Fungal | *Sorbus graudifolia* | Basiodiomycetes: Pucciniaceae |  |
| 275^§^ | cf. *Eriophyes lateannulatus fossilis* |  | Straus (1977; Taf. 4, Fig. 78) |  | 32 | Galling | *Tillia* sp. | Acarina: Eriophyidae |  |
| 7257/a^§^ | *Eriophyes exilis fossilis* vel *Typhlodromus* sp. *fossilis* |  |  |  | 34,16 | Galling | *Tillia* sp. | Acarina: Laelaptidae |  |
| 18059^§^ | *Eriophyes exilis fossilis* vel *Typhlodromus* sp. *fossilis* |  |  |  | 32,33 | Galling | *Tillia* sp. | Acarina: Laelaptidae |  |
| 7087/a^¶,§^ | *Aceria aroniae fossilis* (=*Eriophyes pyri* ) |  |  |  |  | Galling | *Amelanchier* sp. | Acarina: Eriophyidae |  |
| 2614/a^§^ | *Aceria aroniae fossilis* (=*Eriophyes pyri* ) |  |  |  | 32 | Galling | *Crataegus meischnerii* | Acarina: Eriophyidae |  |
| 11661^§^ | *Aceria erinea fossilis* |  | Straus (1977; Taf. 1, Fig. 27) |  | 12,3 | Galling | *Juglans* sp. | Acarina: Eriophyidae |  |
| 18419^§^ | *Aceria erinea fossilis* |  | Straus (1977; Fig. 5) |  | 2 | Galling | *Populus* sp. | Acarina: Eriophyidae |  |
| 9160^¶,§^ | *Aceria erinea fossilis* |  | Straus (1977; Taf. 1, Fig. 26) |  |  | Galling | *Carya* sp./ *Pterocarya* sp. | Acarina: Eriophyidae |  |
| 3201/a^§^ | *Aceria inangulis fossilis* (=*Eriophyes inangulis*) |  | Straus (1977; Fig. 6) |  | 33 | Galling | *Alnus nepalensis* | Acarina: Eriophyidae |  |
| 9159, ^§^ | Aceria leionota fossilis (=*Eriophyes leionotus*) |  | Straus (1977; Taf. 1, Fig. 17) | **Plate B, Fig. A** | 32,3 | Galling | *Betula* sp. | Acarina: Eriophyidae |  |
| 9444/a^§^ | *Aceria leionota fossilis* (=*Eriophyes leionotus*) |  |  | **Plate B, Fig. B** |  | Galling | ?*Carpinus* sp. | Acarina: Eriophyidae |  |
| 17758^¶,§^ | cf. *Aceria macrochela fossilis* |  | Straus (1977; Taf. 2, Fig. 41) |  |  | Galling | *Acer* sp. | Acarina: Eriophyidae |  |
| 3116^§^ | cf. *Aceria macrochela fossilis* |  |  | **Plate B, Fig. C** | ? | Galling | *Acer laetum* | Acarina: Eriophyidae |  |
| 6560^§^ | cf. *Aceria macrochela fossilis* |  | Straus (1977; Taf. 4, Fig. 71) | **Plate B, Fig. D** | 80 | Galling | *Acer laetum* | Acarina: Eriophyidae |  |
| 19779^§^ | cf. *Aceria macrochela fossilis* |  |  | **Plate B, Fig. E** | 32 | Galling | *Acer laetum* | Acarina: Eriophyidae |  |
| 30808^§^ | cf. *Aceria macrochela fossilis* |  |  | **Plate B, Fig. F** | 32 | Galling | *Acer* sp. | Acarina: Eriophyidae |  |
| 30914^§^ | *Aceria nervisequa faginea fossilis* |  | Straus (1977; Taf. 3, Fig. 50) | **Plate B, Fig. G** | 80 | Galling | *Fagus* sp. | Acarina: Eriophyidae |  |
| 21979^§^ | *Aceria nervisequa faginea fossilis* |  | Straus (1977; Fig. 2) | **Plate B, Fig. H** | unidentified | Galling | *Fagus* sp. | Acarina: Eriophyidae |  |
| 30815^§^ | *Aceria nervisequa faginea fossilis* |  |  | **Plate B, Fig. K** | 46 | Galling | *Fagus sylvatica* | Acarina: Eriophyidae |  |
| 30815^¶,*^ | *Phyllocecidium internervellum* | H |  |  |  | Galling | *Fagus* sp. | Acarina: Eriophyidae |  |
| 13748/a^§^ | *Aceria nervisequa nervisequa fossilis* |  | Straus (1977; Taf. 1, Fig. 20) | **Plate B, Fig. L** | unidentified | Galling | *Fagus* cf. *pliocenica* | Acarina: Eriophyidae |  |
| 22848/a^§^ | *Aceria nervisequa nervisequa fossilis* |  |  | **Plate B, Fig. M** | 145 | Galling | *Fagus sylvatica* | Acarina: Eriophyidae |  |
| 38/150^¶,§^ | *Aceria stenaspis stenaspis fossilis* |  | Straus (1977; Fig. 8) |  |  | Galling | *Fagus* sp. | Acarina: Eriophyidae |  |
| 8281^¶,§^ | *Aceria tenella fossilis* |  | Straus (1977; Taf. 3, Fig. 46) |  |  | Galling | *Carpinus* sp. | Acarina: Eriophyidae |  |
| 5778^¶,§^ | *Schizoneura ulmi fossilis* |  |  |  |  | Galling | *Ulmus* sp. | Hemiptera: Eriostomatidae |  |
| 2179^§^ | *Schizoneura ulmi fossilis* |  | Straus (1977; Fig. 4) | **Plate B, Fig. I** | not observed | Galling | *Ulmus* sp. | Hemiptera: Eriostomatidae |  |
| 13419^¶,§^ | *Schizoneura ulmi fossilis* |  | Straus (1977; Taf. 1, Fig. 31) |  |  | Galling | *Ulmus* sp. | Hemiptera: Eriostomatidae |  |
| 23403^¶,§^ | cf. *Dasyneura ruebsaameni fossilis* vel *Cecidophyes reniformis fossilis* |  | Straus (1977; Fig. 1) |  |  | Galling | *Carpinus* sp. | Diptera: Cecidomyiidae vel Acarina: Eriophyidae |  |
| 3765^¶,§^ | cf. *Dasyneura ruebsaameni fossilis* vel *Cecidophyes reniformis fossilis* |  |  |  |  | Galling | *Carpinus* sp. | Diptera: Cecidomyiidae vel Acarina: Eriophyidae |  |
| 23992/a^§^ | cf. *Dasyneura ruebsaameni fossilis* vel *Cecidophyes reniformis fossilis* |  |  | **Plate A, Fig. W** | 32 | Galling | *Carpinus orientalis* | Diptera: Cecidomyiidae vel Acarina: Eriophyidae |  |
| 14652^§^ | cf. *Dasyneura fraxini fossilis* |  | Straus (1977; Taf. 1, Fig. 16) | **Plate B, Fig. J** | 87,1 | Galling | *Fraxinus* sp. | Diptera: Cecidomyiidae |  |
| 18935^¶,§^ | cf. *Myzus* sp. |  | Straus (1977; Taf. 4, Fig. 83) |  |  | Galling | *Prunus* sp. | Hemiptera: Aphididae |  |
| 2928^¶,§^ | cf. *Dreyfusia* sp. *fossilis* |  |  |  |  | Galling | Pinaceae? | Hemiptera: Adelgidae |  |
| 2425^§^ | *Neuroterus albipes fossilis* (= *N. laeviusculus*) |  | Straus (1977; Taf. 2, Fig. 39) | **Plate B, Fig. O** | 32 | Galling | *Quercus praeerucifolia* | Hymenoptera: Cynipidae |  |
| 2737/a^¶,§^ | cf. *Andricus curvator fossilis* |  | Straus (1977; Taf. 1, Fig. 30) |  |  | Galling | *Quercus praeerucifolia* | Hymenoptera: Cynipidae |  |
| 9161^¶,§^ | cf. *Andricus curvator fossilis* |  |  |  |  | Galling | *Quercus praeerucifolia* | Hymenoptera: Cynipidae |  |
| 14200^§^ | *Andricus quercus-radicis fossilis* |  | Straus (1977; Taf. 1, Fig. 18) | **Plate B, Fig. N** | 117 | Galling | *Quercus roburoides* | Hymenoptera: Cynipidae |  |
| 7244^§^ | cf. *Andricus* sp. |  | Straus (1977; Taf.1, Fig. 25) | **Plate B, Fig. S** | 12,32 | Galling | *Quercus roburoides* | Hymenoptera: Cynipidae | *Neuroterus* as possible culprid could not be excluded. Berger (1949) reported comparable galling structures on Pliocene angiosperm leaves |
| 13732^§^ | cf. *Andricus* sp. |  |  | **Plate B, Fig. X** | 32 | Galling | *Quercus praeerucifolia* | Hymenoptera: Cynipidae | *Neuroterus* as possible culprid could not be excluded. Berger (1949) reported comparable galling structures on Pliocene angiosperm leaves |
| 15091^§^ | cf. *Andricus* sp. |  | Straus (1977; Taf. 1, Fig. 21) | **Plate B, Fig. Y** | 32 | Galling | *Quercus praeerucifolia* | Hymenoptera: Cynipidae | *Neuroterus* as possible culprid could not be excluded. Berger (1949) reported comparable galling structures on Pliocene angiosperm leaves |
| 17076^§^ | cf. *Andricus* sp. |  |  | **Plate B, Fig. W** | 32 | Galling | *Quercus praeerucifolia* | Hymenoptera: Cynipidae | *Neuroterus* as possible culprid could not be excluded. Berger (1949) reported comparable galling structures on Pliocene angiosperm leaves |
| 22705^§^ | cf. *Andricus* sp. |  | Straus (1977; Taf. 1, Fig. 24) | **Plate B, Fig. Z** | 142,12 | Galling | *Quercus praeerucifolia* | Hymenoptera: Cynipidae | *Neuroterus* as possible culprid could not be excluded. Berger (1949) reported comparable galling structures on Pliocene angiosperm leaves |
| 5021^§^ | cf. *Mikiola fagi fossilis* vel *Hartigiola annulipes fossilis* |  | Straus (1977; Taf. 1, Fig. 22) | **Plate B, Fig. AA** | unidentified | Galling |  | Diptera: Cecidomyiidae |  |
| 5786^¶,§^ | cf. *Mikiola fagi fossilis* vel *Hartigiola annulipes fossilis* |  |  |  |  | Galling |  | Diptera: Cecidomyiidae |  |
| 8717^§^ | cf. *Didymomyia reaumuriana fossilis* |  | Straus (1977; Fig. 3) | **Plate B, Fig. T** | 78,19 | Galling | *Tillia* sp. | Diptera: Cecidomyiidae |  |
| 8717^*^ | *Fenusites tiliae* | H | Straus (1977; Fig. 3) | **Plate B, Fig. T** | 78,19 | Mining | *Tiliia* sp. | Hymenoptera: Tenthredinidae |  |
| 15650* | *Fenusites tiliae* |  |  | **Plate D, Fig. A** | 2,16 | Mining | *Tilia saportae* | Hymenoptera: Tenthredinidae |  |
| 21695/a^¶,*^ | *Fenusites tiliae* |  |  |  |  | Mining | Tiliia sp. | Hymenoptera: Tenthredinidae |  |
| 22549/a^*^ | *Fenusites tiliae* |  |  | **Plate D, Fig. B** | 2,16 | Mining | *Tilia* cf. *savania* | Hymenoptera: Tenthredinidae |  |
| 3042^§^ | cf. *Contarinia carpini fossilis* |  | Straus (1977; Taf. 1, Fig. 28) | **Plate B, Fig. U** | 32 | Galling | *Populus willershausensis* | Diptera: Cecidomyiidae | Steinbach (1967) and Straus (1962) determined these galls earlier as hymenopteran galls belonging to *Andricus curvator* |
| 8907^¶,§^ | cf. *Phegomyia fagicola fossilis* |  |  |  |  | Galling |  | Diptera: Cecidomyiidae | Steinbach (1967) and Straus (1962) determined these galls earlier as hymenopteran galls belonging to *Andricus curvator* |
| 12110/a^¶,§^ | cf. *Phegomyia fagicola fossilis* |  | Straus (1977; Taf. 2, Fig. 34) |  |  | Galling |  | Diptera: Cecidomyiidae | Steinbach (1967) and Straus (1962) determined these galls earlier as hymenopteran galls belonging to *Andricus curvator* |
| 19960/a^¶,§^ | cf. *Phegomyia fagicola fossilis* |  |  |  |  | Galling |  | Diptera: Cecidomyiidae | Steinbach (1967) and Straus (1962) determined these galls earlier as hymenopteran galls belonging to *Andricus curvator* |
| 30810^*^ | *Petiolocecidium aceris* | H | Straus (1977; Taf. 2, Fig. 38) | **Plate B, Fig. P** | unidentified | Galling | *Acer integerrinum* | Hemiptera: Pemphigidae | Resemble those structures made by *Pemphigus spirotheca* (comp. Mädler, 1936) |
| 20197/a^*^ | *Petioloceoidium hamamelidacearum* | H | Straus (1977; Taf. 2, Fig. 42) | **Plate C, Fig. A** | unidentified | Galling | *Parrotia* sp. |  |  |
| 10060^*^ | *Petioloceoidium hamamelidacearum* |  | Straus (1977; Taf. 1, Fig. 32) | **Plate B, Fig. Q** | 33 | Galling | *Parrotia* sp. |  |  |
| 10959^¶,*^ | *Petioloceoidium hamamelidacearum* |  | Straus (1977; Taf. 1, Fig. 29) |  |  | Galling | cf. *Parrotiopsis* sp. |  |  |
| 13164^§^ | *Petioloceoidium hamamelidacearum* |  |  | **Plate B, Fig. AB** | 16,3 | Galling | *Parrotia persica* |  |  |
| 3388/a^*^ | *Phyllocecidium alni-tuberculosum* | H | Straus (1977; Taf. 2, Fig. 33) | **Plate B, Fig. R** | 32 | Fungal / Galling | *Alnus incana* | Ascomycetes: Exoascaceae vel.  Diptera: Cecidomyiidae |  |
| 12143^*^ | *Phyllocecidium comma* | H | Straus (1977; Taf. 2, Fig. 36) | **Plate B, Fig. V** |  | Galling | *Quercus* sp. |  |  |
| 20161/a^*^ | *Phyllocecidium comma* |  |  | **Plate C, Fig. B** | 32 | Galling | *Quercus praeerucifolia* |  |  |
| 23862^*^ | *Phyllocecidium comma* |  |  | **Plate C, Fig. C** | 32 | Galling | *Quercus praeerucifolia* |  |  |
| 7691^¶,*^ | *Phyllocecidium cuniculatum* | H | Straus (1977; Taf. 3, Fig. 47) |  |  | Galling | cf. *Quercus* |  |  |
| 30815^*^ | *Phyllocecidium internervellum* |  | Straus (1977; Taf. 2, Fig. 43) | **Plate B, Fig. K** | 46 | Galling | *Fagus sylvatica* |  | comp. also with *Hartigiola* and *Aceria nervisequa faginea fossilis* |
| 18421/a^*^ | *Phyllocecidium medionervisequm* | H | Straus (1977; Fig. 9) | **Plate C, Fig. D** | 53 | Galling | *Quercus praeerucifolia* | Hymenoptera: Cynipidae (*Neuroterus* sp.) |  |
| 30845/a^*^ | *Phyllocecidium medionervisequm* |  |  | **Plate C, Fig. E** | 194 | Galling | *Quercus roburoides* |  |  |
| 13156^*^ | *Phyllocecidium parrotiae* | H | Straus (1977; Taf. 4, Fig. 82) | **Plate C, Fig. F** | 145 | Fungal / Galling | Parrotia sp. |  | comparapble to fungal infection caused by *Rhytisma*. Probably also on *Parratiopsis* |
| 2603^*^ | *Phyllocecidium parrotiae* |  |  | **Plate C, Fig. G** | 1 | Fungal / Galling | *Parrotia* sp. |  | comparapble to fungal infection by *Rhytisma*. Probaly also on *Parratiopsis* |
| 7217^*^ | *Phyllocecidium parrotiae* |  |  | **Plate C, Fig. H** | 32 | Fungal / Galling | *Parrotia* sp. |  | comparapble to fungal infection caused by *Rhytisma*. Probably also on *Parratiopsis* |
| 7253^*^ | *Phyllocecidium parrotiae* |  |  | **Plate C, Fig. I** | 210 | Fungal / Galling | *Parrotia* sp. |  | comparapble to fungal infection caused by *Rhytisma*. Probably also on *Parratiopsis* |
| 7411^*^ | *Phyllocecidium parrotiae* |  |  | **Plate C, Fig. J** | 33 | Fungal / Galling | *Parrotia* sp. |  | comparapble to fungal infection caused by *Rhytisma*. Probably also on *Parratiopsis* |
| 9214^*^ | *Phyllocecidium parrotiae* |  | Straus (1977; Taf. 3, Fig. 48) | **Plate C, Fig. K** | 120 | Fungal / Galling | *Parrotia* sp. |  | comparapble to fungal infection caused by *Rhytisma*. Probably also on *Parratiopsis* |
| 9673/a^*^ | *Phyllocecidium parrotiae* |  |  | **Plate C, Fig. L** | 32,17 | Fungal / Galling | *Parrotia* sp. |  | comparapble to fungal infection caused by *Rhytisma*. Probably also on *Parratiopsis* |
| 10196^*^ | *Phyllocecidium parrotiae* |  | Straus (1977; Taf. 2, Fig. 40) | **Plate C, Fig. M** | 2,78 | Fungal / Galling | *Parrotia* sp. |  | comparapble to fungal infection caused by *Rhytisma*. Probably also on *Parratiopsis* |
| 12733^¶,*^ | *Phyllocecidium parrotiae* |  |  |  |  | Fungal / Galling | *Parrotia* sp. |  | comparapble to fungal infection caused by *Rhytisma*. Probably also on *Parratiopsis* |
| 12818^¶,*^ | *Phyllocecidium parrotiae* |  |  |  |  | Fungal / Galling | *Parrotia* sp. |  | comparapble to fungal infection caused by *Rhytisma*. Probably also on *Parratiopsis* |
| 13217^§^ | *Phyllocecidium parrotiae* |  |  | **Plate C, Fig. N** | 146 | Fungal / Galling | *Parrotia* sp. |  | comparapble to fungal infection caused by *Rhytisma*. Probably also on *Parratiopsis* |
| 16732^*^ | *Phyllocecidium parrotiae* |  |  | **Plate C, Fig. O** | 33 | Fungal / Galling | *Parrotia* sp. |  | comparapble to fungal infection caused by *Rhytisma*. Probably also on *Parratiopsis* |
| 17689* | *Phyllocecidium parrotiae* |  |  | **Plate C, Fig. P** | 128 | Fungal / Galling | *Parrotia* sp. |  | comparapble to fungal infection caused by *Rhytisma*. Probably also on *Parratiopsis* |
| 17715^*^ | *Phyllocecidium parrotiae* |  | Straus (1977; Taf. 2, Fig. 37) | **Plate C, Fig. Q** | 33 | Fungal / Galling | *Parrotia* sp. |  | comparapble to fungal infection caused by *Rhytisma*. Probably also on *Parratiopsis* |
| 20318/a^*^ | *Phyllocecidium parrotiae* |  |  | **Plate C, Fig. R** | 12,16 | Fungal / Galling | *Parrotia* sp. |  | comparapble to fungal infection caused by *Rhytisma*. Probably also on *Parratiopsis* |
| 20892^*^ | *Phyllocecidium parrotiae* |  |  | **Plate C, Fig. S** | not observed | Fungal / Galling | *Parrotia* sp. |  | comparapble to fungal infection caused by *Rhytisma*. Probably also on *Parratiopsis* |
| 21920/a^§^ | *Fenusa ulmi fossilis* |  | Straus (1977; Fig. 15) | **Plate C, Fig. U** | 78,2,17 | Mining | *Sorbus gabbrensis* | Hymenoptera: Tenthredinidae | A comparable mining type is also figured on *Ulmus longifolia* by Jakubowskaja (1955) |
| 12^§^ | *Fenusa ulmi fossilis* |  |  | **Plate C, Fig. V** | 78 | Mining | *Fagus* cf. *pliocenica* | Hymenoptera: Tenthredinidae |  |
| 3183^§^ | *Fenusa ulmi fossilis* |  |  | **Plate C, Fig. W** | 78 | Mining | *Ulmus* sp. | Hymenoptera: Tenthredinidae | A comparable mining type is also figured on *Ulmus longifolia* by Jakubowskaja (1955) |
| 30837^¶,§^ | *Fenusa ulmi fossilis* |  |  |  |  | Mining | *Ulmus* sp. | Hymenoptera: Tenthredinidae | A comparable mining type is also figured on *Ulmus longifolia* by Jakubowskaja (1955) |
| 14535/a^*^ | *Fenusites denckmanni* | H | Straus (1977; Taf. 3, Fig. 58) | **Plate C, Fig. X** | 42 | Mining | *Magnolia* sp. vel. *Syringa* sp. | Hymenoptera: Tenthredinidae |  |
| 17885* | *Fenusites denckmanni* |  |  |  | 78 | Mining | *Magnolia* sp. vel. *Syringa* sp. | Hymenoptera: Tenthredinidae |  |
| 20987/a^*^ | *Fenusites betulacearum* | H | Straus (1977; Taf. 3, Fig. 54) | **Plate C, Fig. Y** | 78 | Mining | Betulaceae indet. | Hymenoptera: Tenthredinidae | *Fenusa* type |
| 18874^*^ | *Fenusites betulacearum* |  |  | **Plate C, Fig. T** | 78,21 | Mining | Betulaceae indet. | Hymenoptera: Tenthredinidae | *Fenusa* type |
| 18906^*^ | *Fenusites betulacearum* |  |  | **Plate D, Fig. C** | 32,63 | Mining | Betulaceae indet. | Hymenoptera: Tenthredinidae; Lepidoptera: Coleophoridae | *Fenusa* type |
| 20468/a^¶,*^ | *Fenusites betulacearum +* cf. *Coleophora* sp. |  | Straus (1977; Taf. 2, Fig. 45) |  |  | Mining | Betulaceae indet. | Hymenoptera: Tenthredinidae | *Fenusa* type |
| 17924^*^ | *Fenusites celtis* | H | Straus (1977; Taf. 3, Fig. 53) |  | 21 | Mining | *Celtis* sp. | Hymenoptera: Tenthredinidae | *Fenusa* type |
| 21926^*^ | *Fenusites fagi* | H | Straus (1977; Fig. 13) |  | 15,5 | Mining | *Fagus* sp. | Hymenoptera: Tenthredinidae | *Fenusa* type |
| 11339^*^ | *Fenusites fagi* |  |  |  | 78,16 | Mining | *Fagus* cf. *pliocenica* | Hymenoptera: Tenthredinidae | *Fenusa* type |
| 19782^¶,*^ | *Fenusites fagi* |  |  |  |  | Mining | *Fagus* sp. | Hymenoptera: Tenthredinidae | *Fenusa* type |
| 22508^*^ | *Fenusites parrotiae* | H | Straus (1977; Fig. 11) |  | 16,78 | Mining | *Parrotia* sp. |  | additional frass on the leaf caused by Coleoptera: Hispinae vel. Halticidae |
| 30807^¶,*^ | *Fenusites parrotiae* | P |  |  |  | Mining | *Parrotia* sp. |  |  |
| 30813^¶,*^ | *Fenusites zelkovae* | H | Straus (1977; Taf. 4, Fig. 73) |  |  | Mining | *Zelkova* sp. | Hymenoptera: Tenthredinidae | *Fenusa* type |
| 8068/a^¶,*^ | *Fenusites zelkovae* |  |  |  |  | Mining | *Zelkova* sp. | Hymenoptera: Tenthredinidae | *Fenusa* type |
| 30805^¶,*^ | *Fenusites zelkovae* |  |  |  |  | Mining | *Zelkova* sp. | Hymenoptera: Tenthredinidae | *Fenusa* type |
| 30806^*^ | *Fenusites zelkovae* |  |  |  |  | Mining | *Quercus praeerucifolia* | Hymenoptera: Tenthredinidae | *Fenusa* type |
| 30814/a^¶,§^ | cf. *Profenusa pygmaea fossilis* |  |  |  |  | Mining | *Quercus petrae* vel. *Quercus iberica* | Hymenoptera: Tenthredinidae | *Fenusa* type |
| 19783^¶,§^ | cf. *Profenusa pygmaea fossilis* |  |  |  |  | Mining | *Quercus petrae* vel. *Quercus iberica* | Hymenoptera: Tenthredinidae | *Fenusa* type |
| 18429 vel 18422^§^ | cf. *Bucculatrix thoracella fossilis* |  | Straus (1977; Taf. 3, Fig. 61) |  | 12,32 | Mining | *Ampelopsis cordataeformis* | Lepidoptera: Lyonetiidae |  |
| 21040^§^ | cf. *Coleophora* sp. |  |  |  | 5 | Mining | *Betula maximowicziana* | Lepidoptera: Coleophoridae |  |
| 21695/a^§^ | cf. *Coleophora* sp. |  |  |  | 4,5, 80 | Mining | *Tilia* sp. | Lepidoptera: Coleophoridae |  |
| 22549/a^§^ | cf. *Coleophora* sp. |  |  |  | 2,16 | Mining |  | Lepidoptera: Coleophoridae |  |
| 22858^§^ | cf. *Coleophora* sp. |  | Straus (1977; Taf. 3, Fig. 56) |  |  | Mining |  | Lepidoptera: Coleophoridae |  |
| 22907^¶,§^ | cf. *Coleophora* sp. |  |  |  |  | Mining |  | Lepidoptera: Coleophoridae |  |
| 22996/a^§^ | cf. *Coleophora* sp. |  |  |  | 3 | Mining |  | Lepidoptera: Coleophoridae |  |
| 30809^¶,§^ | cf. *Coleophora* sp. |  |  |  |  | Mining |  | Lepidoptera: Coleophoridae |  |
| 22788^§^ | cf. *Caloptilia alchimiella fossilis* |  |  |  | 1,3 | Mining | *Fagus* cf. *orientalis* | Lepidoptera: Gracilariidae |  |
| 22440^§^ | cf. *Caloptilia roscipenella fossilis* |  | Straus (1977; Taf. 4, Fig. 76) |  | 152,  21 | Mining | *Fagus* sp. | Lepidoptera: Gracilariidae |  |
| 30838^§^ | cf. *Coriscium* sp. |  | Straus (1977; Taf. 3, Fig. 60) |  | 142 | Mining | *Magnolia* sp. vel. *Syringa* sp. | Lepidoptera: Gracilariidae |  |
| 15876/a^§^ | cf. *Parornix* sp. |  | Straus (1977; Taf. 3, Fig. 49) |  | 15 | Mining | *Carpinus orientalis* | Lepidoptera: Gracilariidae |  |
| 30057^§^ | *Lithocolletis maestingella fossilis* |  | Straus (1977; Taf. 3, Fig. 59) |  |  | Mining | *Fagus* sp. | Lepidoptera: Gracilariidae |  |
| 15026^§^ | *Lithocolletis maestingella fossilis* |  |  |  |  | Mining | *Fagus* sp. | Lepidoptera: Gracilariidae |  |
| 15427^§^ | cf. *Incurvaria oehlmanniella fossilis* |  | Straus (1977; Taf. 2, Fig. 44) |  | 36 | Mining | cf. *Vaccinium* sp. | Lepidoptera: Incurvariidae |  |
| 21313^§^ | cf. *Incurvaria* sp. |  | Straus (1977; Taf. 3, Fig. 55) |  | 32,7 | Mining | *Berberis* sp. | Lepidoptera: Incurvariidae | comp. Hering (1957: 166; Nr. 770) |
| 12724/a^§^ | cf. *Recurvaria nanella* |  | Straus (1977; Taf. 3, Fig. 51) |  | 32 | Mining | *Sorbus torminalia fossilis* | Lepidoptera: Gelechiidae |  |
| 3050^¶,*^ | *Stigmella pliotityrella* | H |  |  |  | Mining | *Fagus sylvatica* | Lepidoptera: Nepticulidae | Holotype described by Kernbach (1967: 105-106, Fig. 4) |
| 9111^§^ | *Stigmella ulmivora fossilis* |  |  |  |  | Mining | *Ulmus* sp. | Lepidoptera: Nepticulidae | comp. Kernbach (1967: 105-106, Fig. 5) |
| 17738^§^ | *Stigmella ulmivora fossilis* |  | Straus (1977; Fig. 12) | **Plate E, Fig. A** | 36 | Mining | ?*Sorbus* *domestica* | Lepidoptera: Nepticulidae |  |
| 22763^§^ | *Stigmellites carpini-orientalis* | H | Straus (1977; Taf. 4, Fig. 80) | **Plate E, Fig. B** | 94 | Mining | Carpinus sp. | Lepidoptera: Nepticulidae |  |
| 22134^§^ | *Stigmellites carpini-orientalis* | P | Straus (1977; Taf. 3, Fig. 62) | **Plate E, Fig. C** | 94 | Mining | Carpinus sp. | Lepidoptera: Nepticulidae |  |
| 11137^¶,*^ | *Stigmellites heringi* | H |  |  |  | Mining | Berberis sp. | Lepidoptera: Nepticulidae | Holotype described by Kernbach (1967: 104-106, Fig. 3) |
| 23973^¶,*^ | *Stigmellites zekovae* | H | Straus (1977; Fig. 14) |  |  | Mining | Zelkova sp. | Lepidoptera: Nepticulidae |  |
| 19753/a | Diptera mine undetermined |  |  | **Plate E, Fig. F** | 90 | Mining | Fraxinus sp. Fraxinus ornus | Diptera vel Coleoptera | most probably these mines belong to mines produced by Diptera larvae (comp. Hering (1957: 456; Nr. 2242)). Alternatively, comparable mines could also be produced by weevils (Coleoptera: Curculionidae) from the genus *Steronychus* feeding on *Fraxinus* sp. |
| 8764/a^¶,§^ | *Phytomya ranunculi fossilis* |  |  |  |  | Mining | ?*Ranunculus repens* | Diptera: Agromyzidae | see also discussion in Winkler et al. (2010) |
| 10965^§^ | *Phytomyzites corni* | H | Straus (1977; Taf. 3, Fig. 52) | **Plate E, Fig. D** | 5 | Mining | Fabaceae | Diptera: Agromyzidae |  |
| 30816^¶,§^ | cf. *Phytagromyza populicola fossilis* | H |  |  |  | Mining | Populus latior | Diptera: Agromyzidae |  |
| 30818^¶,§^ | cf. *Phytagromyza populicola fossilis* |  | Straus (1977; Taf. 4, Fig. 75) |  |  | Mining | Populus latior | Diptera: Agromyzidae vel Coleoptera: Curcuionidae | this mine could also be produced by *Rhynchaenus* cf. populi (Coleoptera: Curculionidae) |
| 19471^§^ | *Cuniculonomus carpini* | H | Straus (1977; Taf. 3, Fig. 57) | **Plate E, Fig. E** | 185 | Mining | *Carpinus betulus* |  | comp. Engelhardt (1876) and Kinzelbach (1970) |
| 30836^¶,§^ | *Cuniculonomus carpini* | P | Straus (1977; Taf. 4, Fig. 79) |  |  | Mining |  | Coleoptera: Curculionidae (*Rhychaenus* sp.) |  |
| 30607^¶,§^ | *Loconomus vitis* | H | Straus (1977; Taf. 4, Fig. 66, 77) |  |  | Mining | *Vitis* sp. |  | blotch mine habitus |
| 30821^¶^ | *Loconomus* sp. |  | Straus (1977; Taf. 4, Fig. 81) |  |  | Mining | cf. *Toona* | ?Diptera: Agrpmyzidae (*Agromyza*, *Phytagromyza*, *Dibolia*) | comp. To mines produced by Agromyza, Phytagromyza, Dibolia |
| 2993/a* | *Phagophytichnus catellarius* | H | Straus (1977; Fig. 10) | **Plate A, Fig. T** | 297 | Skeletonization | Parrotia persica | Coleoptera | produced by Coleoptera: Chrysomelidae: Halticinae and/or Hispinae |
| 10626* | *Phagophytichnus catellarius* | P | Straus (1977; Fig. 7) | **Plate A, Fig. U** | 297 | Skeletonization | *Parrotia persica* | Coleoptera | produced by Coleoptera: Chrysomelidae: Halticinae and/or Hispinae |
| 13076* | *Phagophytichnus catellarius* |  |  | **Plate A, Fig. V** | 298 | Skeletonization | *Parrotia persica* | Coleoptera | produced by Coleoptera: Chrysomelidae: Halticinae and/or Hispinae |
| 12917* | *Phagophytichnus circumsecans* |  |  | **Plate A, Fig. A** | 1,297 | Hole | *Parrotia persica* | Coleoptera? | interpreted by Straus (1977) as the intitial phase (oviposition site) of *Phagophytichnus catellarius* |
| 21482* | *Phagophytichnus nervillos-reliquens* | H | Straus (1977; Taf. 4, Fig. 69) | **Plate A, Fig. Q** | 17 | Skeletonization | *Parrotia persica* | ?Coleoptera vel Hymenoptera |  |
| 7612^¶,*^ | *Phagophytichnus nervillos-reliquens* |  |  |  |  | Skeletonization |  |  |  |
| 11490/a* | *Phagophytichnus nervillos-reliquens* |  |  | **Plate A, Fig. C** | 17,12,2 | Skeletonization |  | ?Coleoptera vel Hymenoptera |  |
| 21140* | *Phagophytichnus nervillos-reliquens* |  |  | **Plate A, Fig. S** | 17 | Skeletonization |  | ?Coleoptera vel Hymenoptera |  |
| 30892/a* | *Phagophytichnus nervillos-reliquens* |  |  | **Plate A, Fig. R** | 17 | Skeletonization | *Parrotia persica* |  |  |
| 11490* | *Phagophytichnus marginis-folii* |  |  | **Plate A, Fig. C** | 17,12,2 | Margin | *Quercus* sp. |  |  |
| 11490* | *Phagophytichnus circumsecans* |  |  | **Plate A, Fig. C** | 2 | Hole | *Quercus* sp. |  |  |
| 12231a* | *Phagophytichnus circumsecans + (Phagophytichnus catellarius)* | H | Straus (1977; Taf. 4, Fig. 70) | **Plate A, Fig. B** | 17,2 | Hole | *Parrotia persica* | Coleoptera? | Coleoptera: Curculionidae or Coleoptera: Chrysomelidae are able to produce such pattern |
| 13386* | *Phagophytichnus marginis-folii* | H |  | **Plate A, Fig. E** | 297 | Margin | *Parrotia persica* | ?Coleoptera: Curculionidae vel Chrysomelidae | Coleoptera: Curculionidae (*Rhynchaenus* or *Otiorhynchus*) or Coleoptera: Chrysomelidae (larvae of Halticinae or Hispinae) are able to produce such pattern |
| 2132/a* | *Phagophytichnus marginis-folii* |  |  | **Plate A, Fig. F** | 297 | Margin | *Parrotia persica* | ?Coleoptera: Curculionidae vel Chrysomelidae | Coleoptera: Curculionidae (*Rhynchaenus* or *Otiorhynchus*) or Coleoptera: Chrysomelidae (larvae of Halticinae or Hispinae) are able to produce such pattern |
| 3532* | *Phagophytichnus marginis-folii* |  | Straus (1977; Taf. 4, Fig. 67) | **Plate A, Fig. D** | unidentified | Margin |  | ?Coleoptera: Curculionidae vel Chrysomelidae | Coleoptera: Curculionidae (*Rhynchaenus* or *Otiorhynchus*) or Coleoptera: Chrysomelidae (larvae of Halticinae or Hispinae) are able to produce such pattern |
| 3612* | *Phagophytichnus marginis-folii* |  |  | **Plate A, Fig. H** | 1 | Margin | *Fraxinus* sp | ?Coleoptera: Curculionidae vel Chrysomelidae | Coleoptera: Curculionidae (*Rhynchaenus* or *Otiorhynchus*) or Coleoptera: Chrysomelidae (larvae of Halticinae or Hispinae) are able to produce such pattern |
| 7227* | *Phagophytichnus marginis-folii* |  |  | **Plate A, Fig. G** | 81,16 | Margin | *Cedrela heliconia* | ?Coleoptera: Curculionidae vel Chrysomelidae | Coleoptera: Curculionidae (*Rhynchaenus* or *Otiorhynchus*) or Coleoptera: Chrysomelidae (larvae of Halticinae or Hispinae) are able to produce such pattern |
| 13298* | *Phagophytichnus marginis-folii* |  |  | **Plate A, Fig. J** | 15,1 | Margin |  | ?Coleoptera: Curculionidae vel Chrysomelidae | Coleoptera: Curculionidae (*Rhynchaenus* or *Otiorhynchus*) or Coleoptera: Chrysomelidae (larvae of Halticinae or Hispinae) are able to produce such pattern |
| 20544* | *Phagophytichnus marginis-folii* |  | Straus (1977; Taf. 4, Fig. 64) | **Plate A, Fig. K** | 15,1 | Margin |  | ?Coleoptera: Curculionidae vel Chrysomelidae | Coleoptera: Curculionidae (*Rhynchaenus* or *Otiorhynchus*) or Coleoptera: Chrysomelidae (larvae of Halticinae or Hispinae) are able to produce such pattern |
| 20615^¶,*^ | *Phagophytichnus marginis-folii* |  |  |  |  | Margin |  | ?Coleoptera: Curcu,lionidae vel Chrysomelidae | Coleoptera: Curculionidae (*Rhynchaenus* or *Otiorhynchus*) or Coleoptera: Chrysomelidae (larvae of Halticinae or Hispinae) are able to produce such pattern |
| 21266* | *Phagophytichnus marginis-folii* |  |  | **Plate A, Fig. I** | 12,1 | Margin | *Fraxinus ornus* | ?Coleoptera: Curculionidae vel Chrysomelidae | Coleoptera: Curculionidae (*Rhynchaenus* or *Otiorhynchus*) or Coleoptera: Chrysomelidae (larvae of Halticinae or Hispinae) are able to produce such pattern |
| 22686* | *Phagophytichnus marginis-folii* |  | Straus (1977; Taf. 4, Fig. 65) | **Plate A, Fig. L** | 15 | Margin | *Parrotia persica* | ?Coleoptera: Curculionidae vel Chrysomelidae | Coleoptera: Curculionidae (*Rhynchaenus* or *Otiorhynchus*) or Coleoptera: Chrysomelidae (larvae of Halticinae or Hispinae) are able to produce such pattern |
| 30811^¶,*^ | *Phagophytichnus marginis-folii* |  |  |  |  | Margin |  | ?Coleoptera: Curculionidae vel Chrysomelidae | Coleoptera: Curculionidae (*Rhynchaenus* or *Otiorhynchus*) or Coleoptera: Chrysomelidae (larvae of Halticinae or Hispinae) are able to produce such pattern |
| 30819* | *Phagophytichnus marginis-folii* |  | Straus (1977; Taf. 4, Fig. 72) | **Plate A, Fig. N** | 14,8 | Margin | *Fraxinus ornus* | ?Coleoptera: Curculionidae vel Chrysomelidae | Coleoptera: Curculionidae (*Rhynchaenus* or *Otiorhynchus*) or Coleoptera: Chrysomelidae (larvae of Halticinae or Hispinae) are able to produce such pattern |
| 30955* | *Phagophytichnus marginis-folii* |  |  | **Plate A, Fig. M** | 12 | Margin | *Tilia saportaea* | ?Coleoptera: Curculionidae vel Chrysomelidae | Coleoptera: Curculionidae (*Rhynchaenus* or *Otiorhynchus*) or Coleoptera: Chrysomelidae (larvae of Halticinae or Hispinae) are able to produce such pattern |
| 11710* | *Phagophytichnus nervos-mutans* | H | Straus (1977; Taf. 4, Fig. 63) | **Plate A, Fig. O** | 12,32 | Margin | *Carya minor* |  |  |
| 9781* | *Phagophytichnus nigromarginatus* | H | Straus (1977; Taf. 4, Fig. 68) | **Plate A, Fig. P** | 8 | Hole | *Laburnum* sp. | ?Coleoptera: Chrysomelidae | comparable to *Phagophytichnus catellarius* |


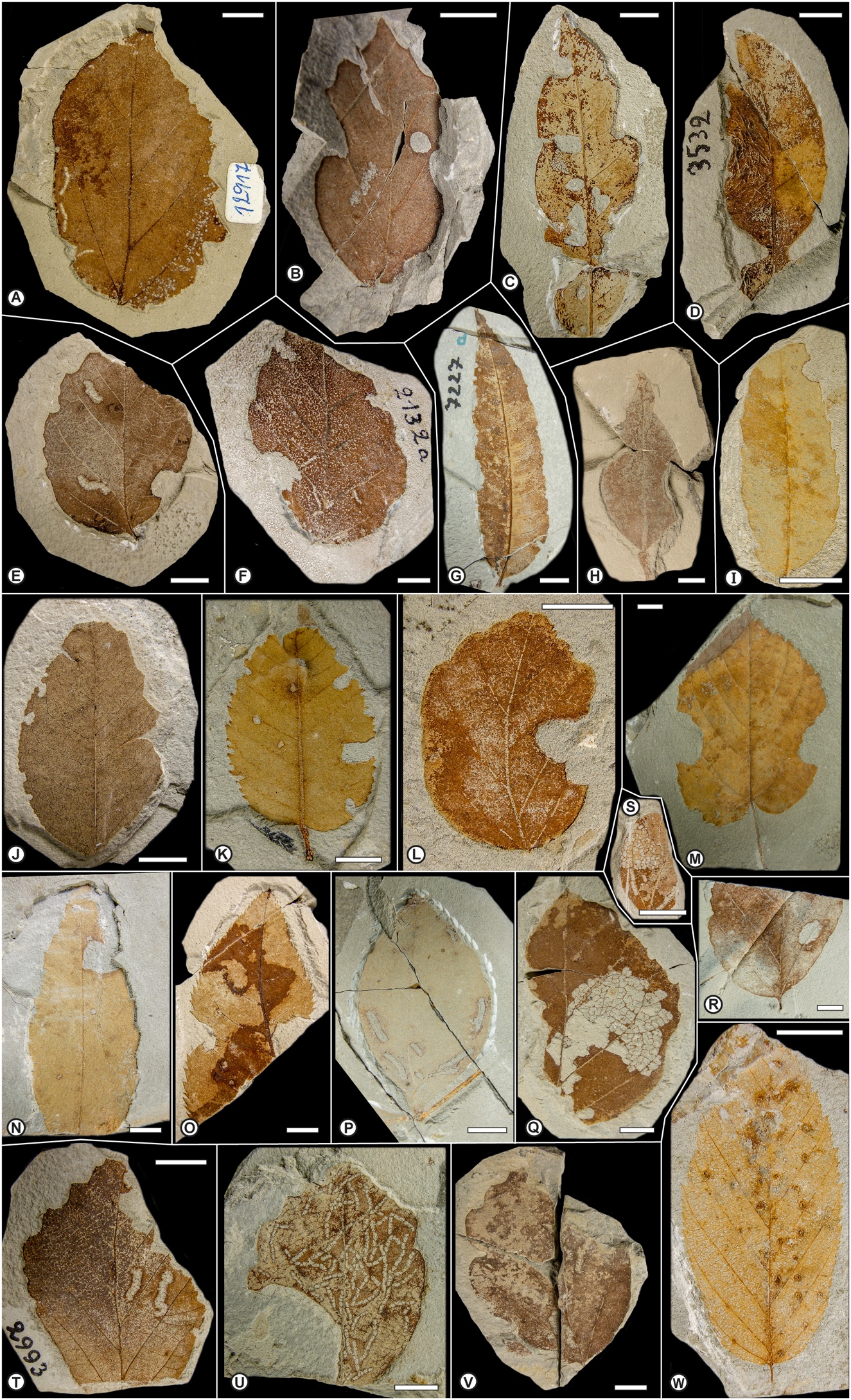


**Plate A.** Types of hervivorized leaves as described by A. Strauss (1977) from the Pliocene of Willershausen (type material is stored in the Willershausen locality, showing herbivory pattern. The material is deposited in the Geoscience Centre, University of Göttingen (GZG). **A.** *Phagophytichnus circumsecans* / *Phagophytichnus catellarius* (GZG.W 12917); **B.** *Phagophytichnus circumsecans* (Holotype, GZG.W 12231); **C.** *Phagophytichnus marginis-folii* / *Phagophytichnus circumsecans* (GZG.W 11490); **D.** *Phagophytichnus marginis-folii* (GZG.W 3532); **E.** *Phagophytichnus marginis-folii* (Holotype, GZG.W 13386); **F.** *Phagophytichnus marginis-folii* (GZG.W 2132/a); **G.** *Phagophytichnus marginis-folii* (GZG.W 7227); **H.** *Phagophytichnus marginis-folii* (GZG.W 3612); **I.** *Phagophytichnus marginis-folii* (GZG.W 21266); **J.** *Phagophytichnus marginis-folii* (GZG.W 13298); **K.** *Phagophytichnus marginis-folii* (GZG.W 20544); **L.** *Phagophytichnus marginis-folii* (GZG.W 22686); **M.** *Phagophytichnus marginis-folii* (GZG.W 30955); **N.** *Phagophytichnus marginis-folii* (GZG.W 30819); **O.** *Phagophytichnus nervos-mutans* (Holotype, GZG.W 11710); **P.** *Phagophytichnus nigromarginatus* (Holotype, GZG.W 9781); **Q.** *Phagophytichnus nervillos-reliquens* (Holotype, GZG.W 21482); **R.** *Phagophytichnus nervillos-reliquens* (GZG.W 30892/a); **S.** *Phagophytichnus nervillos-reliquens* (GZG.W 21140); **T.** *Phagophytichnus catellarius* (Holotype, GZG.W 2993/a); **U.** *Phagophytichnus catellarius* (Paratype, GZG.W 10626); **V.** *Phagophytichnus catellarius* (GZG.W 13076); **W.** cf. *Dasyneura ruebsaameni fossilis* vel *Cecidophyes reniformis fossilis* (GZG.W 23992/a). Scale bars represent 10mm.


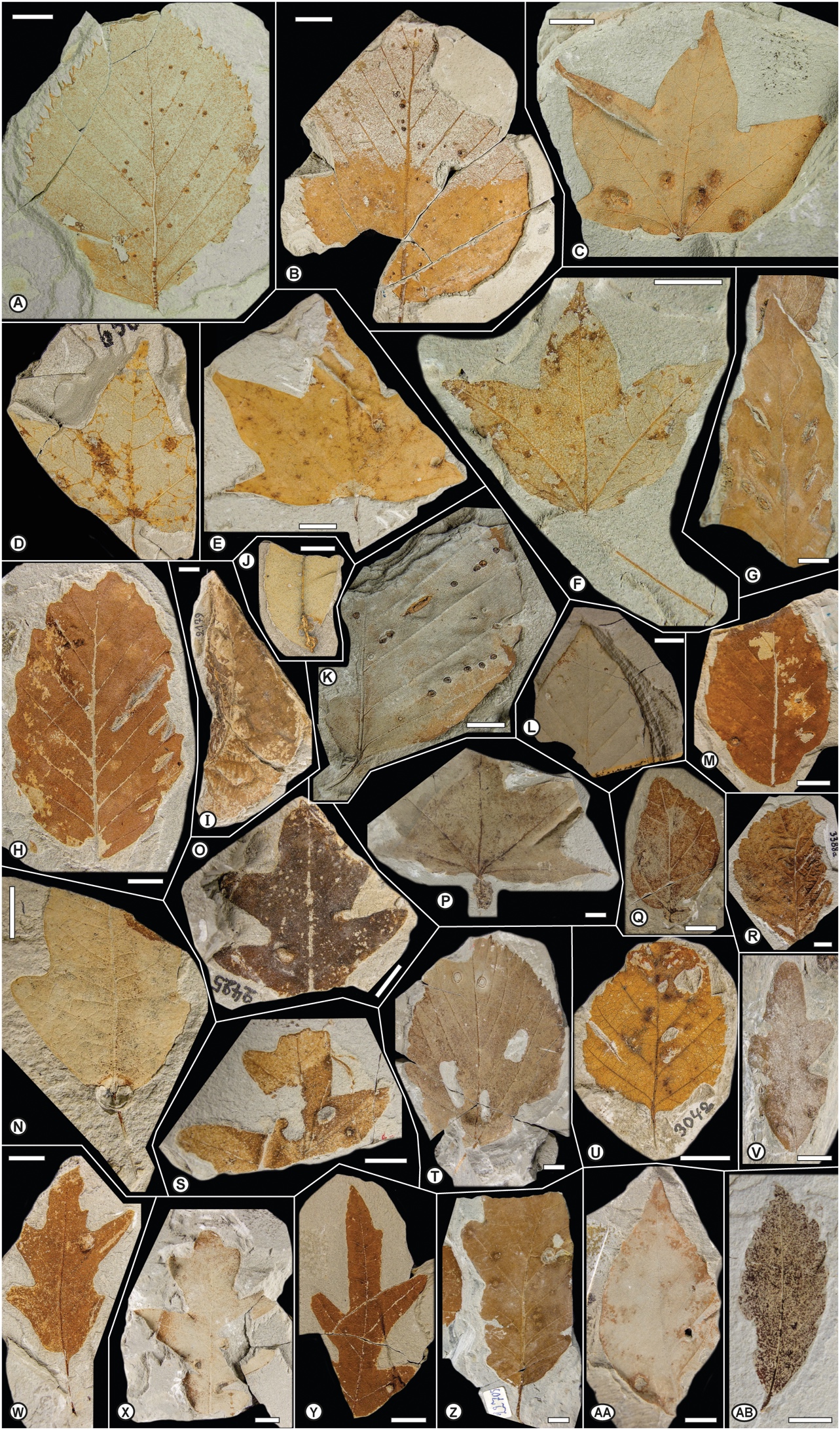


**Plate B.** Types of hervivorized leaves as described by A. Strauss (1977) from the Pliocene of Willershausen (type material is stored in the Willershausen locality, showing herbivory pattern. The material is deposited in the Geoscience Centre, University of Göttingen (GZG). **A.** *Aceria leionota fossilis* (GZG.W 9159); **B.** *Aceria leionota fossilis* (GZG.W 9444/a); **C.** cf. *Aceria macrochela fossilis* (GZG.W 3116); **D.** cf. *Aceria macrochela fossilis* (GZG.W 6560); **E.** cf. *Aceria macrochela fossilis* (GZG.W 19779); **F.** cf. *Aceria macrochela fossilis* (GZG.W 30808); **G.** *Aceria nervisequa faginea fossilis* (GZG.W 30914); **H.** *Aceria nervisequa faginea fossilis* (GZG.W 21979); **I.** *Schizoneura ulmi fossilis* (GZG.W 2179); **J.** cf. *Dasyneura fraxini fossilis* (GZG.W 14652); **K.** *Aceria nervisequa faginea fossilis* (GZG.W 30815); **L.** *Aceria nervisequa nervisequa fossilis* (GZG.W 13748/a); **M.** *Aceria nervisequa nervisequa fossilis* (GZG.W 22848/a); **N.** *Andricus quercus-radicis fossilis* (GZG.W 14200); **O.** *Neuroterus albipes fossilis* (GZG.W 2425); **P.** *Petiolocecidium aceris* (Holotype, GZG.W 30810); **Q.** *Petioloceoidium hamamelidacearum* (GZG.W 10060); **R.** *Phyllocecidium alni-tuberculosum* (Holotype, GZG.W 3388/a); **S.** cf. *Andricus* sp. (GZG.W 7244); **T.** cf. *Didymomyia reaumuriana fossilis* (GZG.W 8717) / *Fenusites tiliae* (Holotype, GZG.W 8717); **U.** cf. *Contarinia carpini fossilis* (GZG.W 3042); **V.** *Phyllocecidium comma* (Holotype, GZG.W 12143); **W.** cf. *Andricus* sp. (GZG.W 17076); **X.** cf. *Andricus* sp. (GZG.W 13732); **Y.** cf. *Andricus* sp. (GZG.W 15091); **Z.** cf. *Andricus* sp. (GZG.W 22705); **AA.** cf. *Mikiola fagi fossilis* vel *Hartigiola annulipes fossilis* (GZG.W 5021); **AB.** *Petioloceoidium hamamelidacearum* (GZG.W 13164). Scale bars represent 10mm.


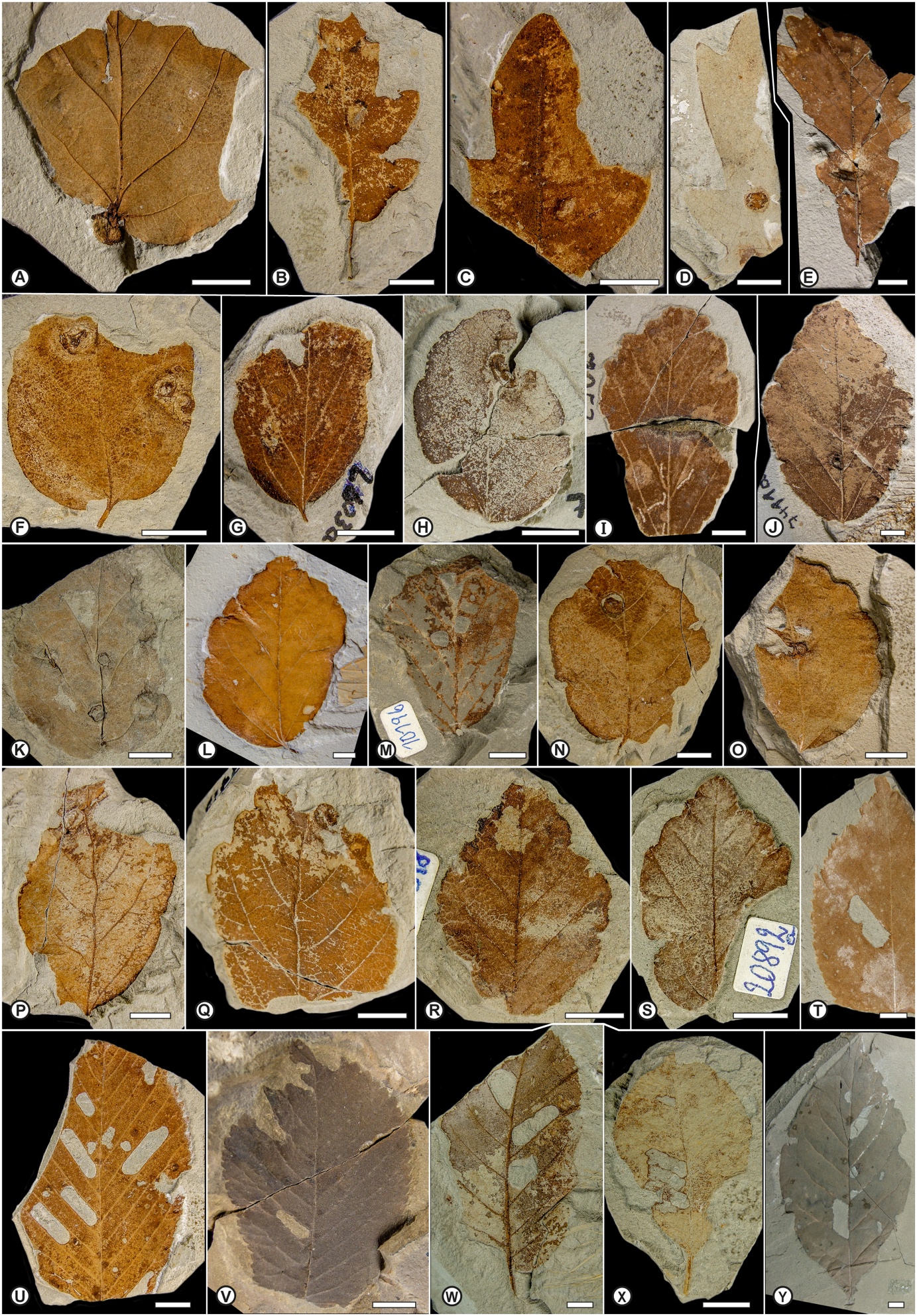


**Plate C.** Types of hervivorized leaves as described by A. Strauss (1977) from the Pliocene of Willershausen (type material is stored in the Willershausen locality, showing herbivory pattern. The material is deposited in the Geoscience Centre, University of Göttingen (GZG). **A.** *Petioloceoidium hamamelidacearum* (Holotype, GZG.W 20197/a); **B.** *Phyllocecidium comma* (Paratype, GZG.W 20161/a); **C.** *Phyllocecidium comma* (Paratype, GZG.W 23862); **D.** *Phyllocecidium medionervisequm* (Holotype, GZG.W 18421/a); **E.** *Phyllocecidium medionervisequm* (GZG.W 30845/a); **F.** *Phyllocecidium parrotiae* (Holotype, GZG.W 13156); **G.** *Phyllocecidium parrotiae* (GZG.W 2603); **H.** *Phyllocecidium parrotiae* (GZG.W 7217); **I.** *Phyllocecidium parrotiae* (GZG.W 7253); **J.** *Phyllocecidium parrotiae* (GZG.W 7411); **K.** *Phyllocecidium parrotiae* (GZG.W 9214); **L.** *Phyllocecidium parrotiae* (GZG.W 9673/a); **M.** *Phyllocecidium parrotiae* (GZG.W 10196); **N.** *Phyllocecidium parrotiae* (GZG.W 13217); **O.** *Phyllocecidium parrotiae* (GZG.W 16732); **P.** *Phyllocecidium parrotiae* (GZG.W 17689); **Q.** *Phyllocecidium parrotiae* (GZG.W 17715); **R.** *Phyllocecidium parrotiae* (GZG.W 20316/a); **S.** *Phyllocecidium parrotiae* (GZG.W 20892); **T.** *Fenusites betulacearum* (GZG.W 18874); **U.** *Fenusa ulmi fossilis* (GZG.W 21920/a); **V.** *Fenusa ulmi fossilis* (GZG.W 12); **W.** *Fenusa ulmi fossilis* (GZG.W 3183); **X.** *Fenusites denckmanni* (Holotype, GZG.W 14535/a); **Y.** *Fenusites betulacearum* (Holotype, GZG.W 20987/a). Scale bars represent 10mm.


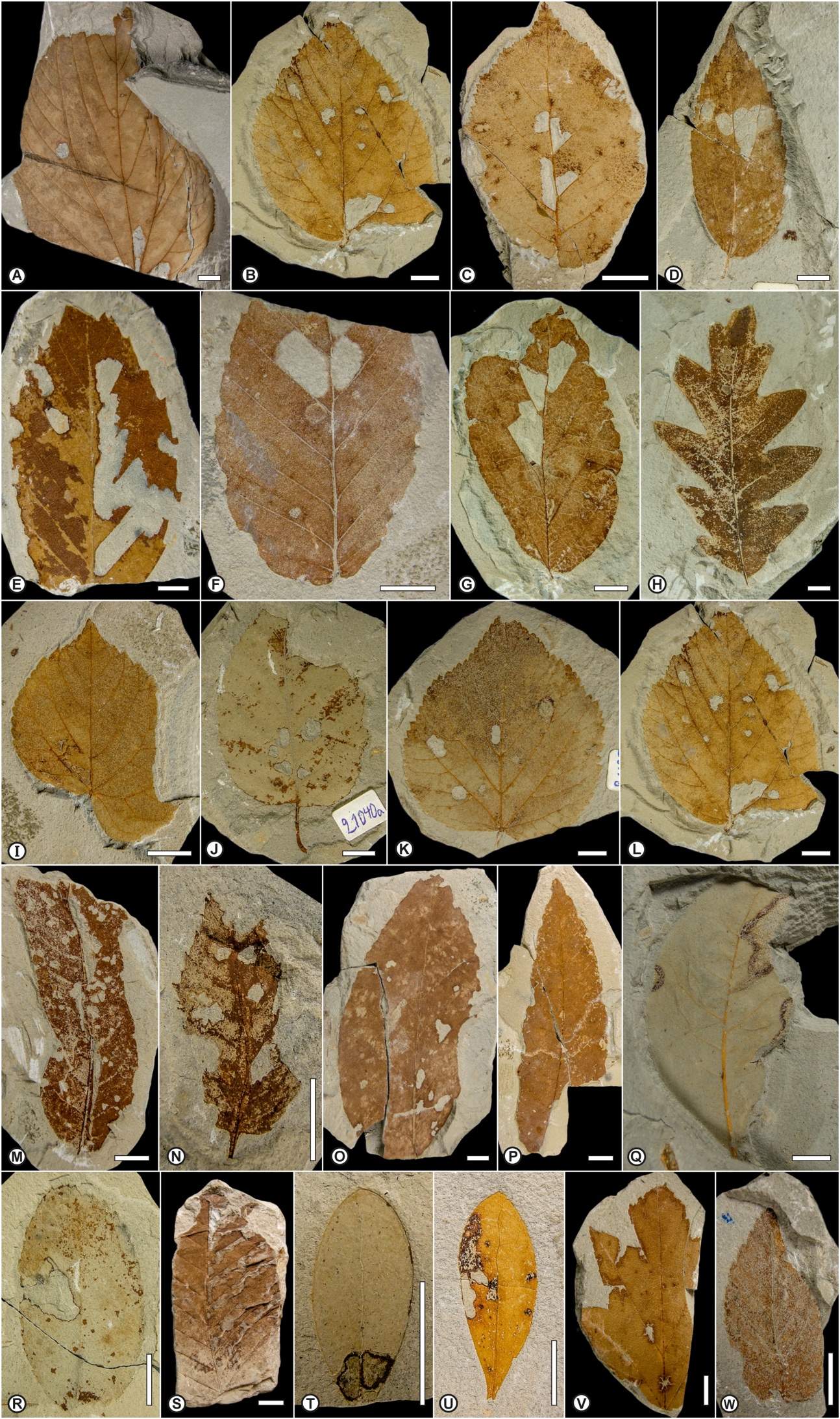


**Plate D.** Types of hervivorized leaves as described by A. Strauss (1977) from the Pliocene of Willershausen (type material is stored in the Willershausen locality, showing herbivory pattern. The material is deposited in the Geoscience Centre, University of Göttingen (GZG). **A.** *Fenusites tiliae* (GZG.W 15650); **B.** *Fenusites tiliae* and cf. *Coleophora* sp. (GZG.W 22549/a); **C.** *Fenusites betulacearum* (GZG.W 18906); **D.** *Fenusites celtis* (Holotype, GZG.W 17924); **E.** *Fenusites fagi* (Holotype, GZG.W 21926); **F.** *Fenusites fagi* (GZG.W 11339); **G.** *Fenusites parrotiae* (Holotype, GZG.W 22508); **H.** *Fenusites zelovae* (Holotype, GZG.W 30806); **I.** cf. *Bucculatrix thoracella fossilis* (GZG.W 18422); **J.** cf. *Coleophora* sp. (GZG.W 21040); **K.** cf. *Coleophora* sp. (GZG.W 21695/a); **L.** cf. *Coleophora* sp. (GZG.W 22549/a); **M.** cf. *Coleophora* sp. (GZG.W 22858); **N.** cf. *Coleophora* sp. (GZG.W 22996/a); **O.** cf. *Caloptilia alchimiella fossilis* (GZG.W 22788); **P.** cf. *Caloptilia alchimiella fossilis* (GZG.W 22440); **Q.** cf. *Coriscium* sp. (GZG.W 30838); **R.** cf. *Parornix* sp. (GZG.W 15876/a); **S.** *Lithocolletis maestingella fossilis* (GZG.W 30057); **T.** cf. *Incurvaria oehlmanniella fossilis* (GZG.W 15427); **U.** cf. *Incurvaria* sp. (GZG.W 21313); **V.** cf. *Recurvaria nanella* (GZG.W 12724/a); W. *Stigmella ulmivora fossilis* (GZG.W 9111). Scale bars represent 10mm.


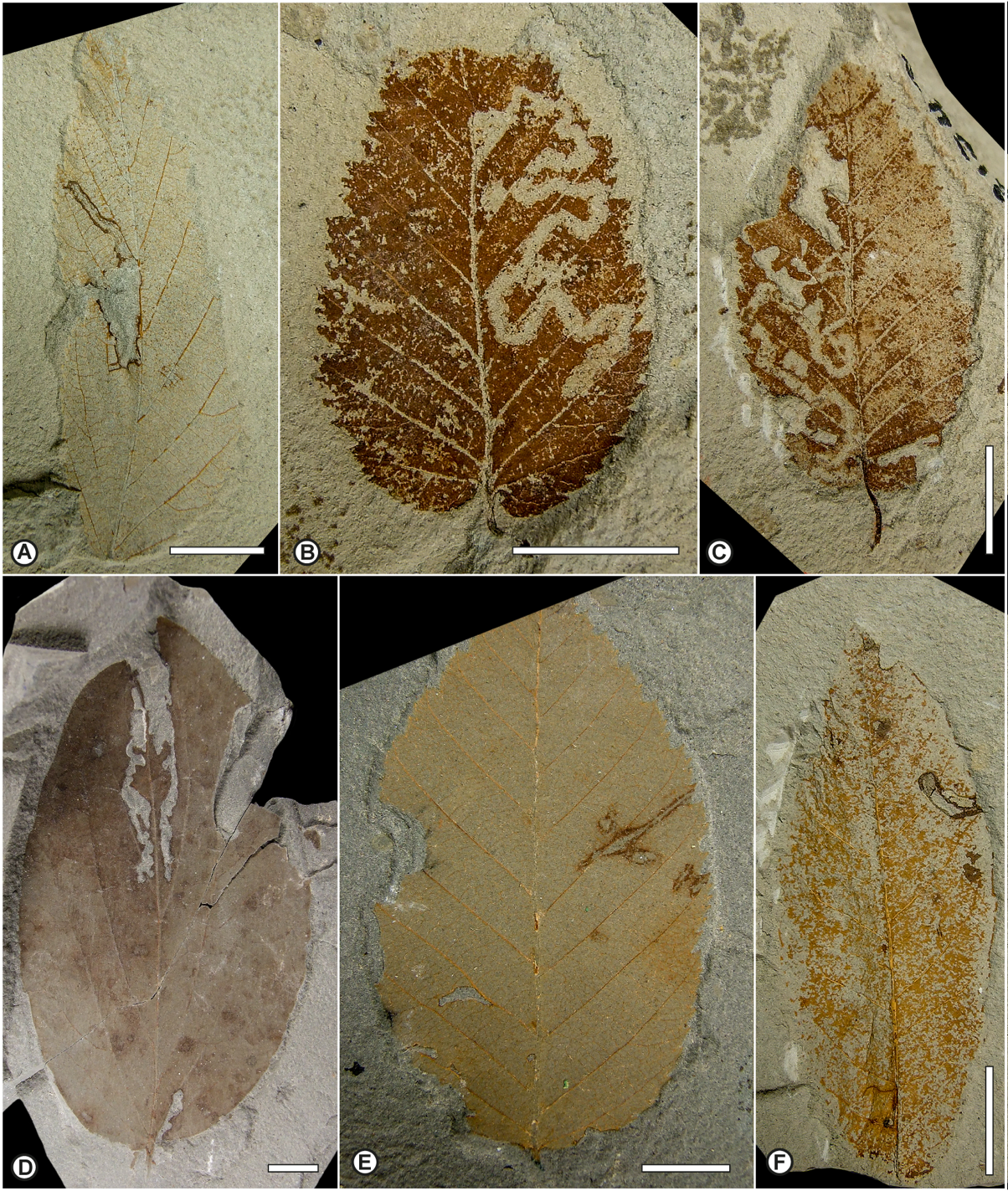


**Plate E.** Types of hervivorized leaves as described by A. Strauss (1977) from the Pliocene of Willershausen (type material is stored in the Willershausen locality, showing herbivory pattern. The material is deposited in the Geoscience Centre, University of Göttingen (GZG). **A.** *Stigmella ulmivora fossilis* (GZG.W 17738); **B.** *Stigmellites carpini-orientalis* (Holotype, GZG.W 22763); **C.** *Stigmellites carpini-orientalis* (Paratype, GZG.W 22134); **D.** *Phytomyzites corni* (Holotype, GZG.W 10965); **E.** *Cuniculonomus carpini* (Holotype, GZG.W 19471); **F.** Diptera mine undetermined (GZG.W 19753/a). Scale bars represent 10mm.

**References**

**Berger W. 1949.** Lebensspuren schmarotzender Insekten an jungtertiären Laubblättern. *Sitzungsberichte der Österreichische Akademie der Wissenschaften, Mathematisch-naturwissenschaftliche Klasse, Sitzungsberichte Abteilung I, Biologie, Mineralogie, Erdkunde und verwandte Wissenschaften* **158**:789–792.

**Bertling M, Braddy S, Bromley R, Demathieu G, Genise J, Mikuláš R, Nielsen J, Nielsen K, Rindsberg A, Schlirf M, Uchman A. 2006.** Names for trace fossils: a uniform approach. *Lethaia* **39**:265–286.

**Buhr H. 1964-1965.** *Bestimmungstabellen der Gallen (Zoo- und Phytocecidien) an Pflanzen Mittel- und Nordeuropas. Band I-II*. Jena: VEB Gustav Fischer Verlag.

**Cariglino B, Gutiérrez PR. 2011.** Plant-insect interactions in a *Glossopteris* flora from the La Golondrina Formation (Guadalupian–Lopingian), Santa Cruz Province, Patagonia, Argentina. *Ameghiniana* **48**:103–112.

**Dieguez C, Nieves-Aldrey J, Barron E. 1996.** Fossil galls (zoocecids) from the Upper Miocene of La Cerdana (Lerida, Spain). *Review of Palaeobotany and Palynology* **94**:329–343.

**Doorenweerd C, Nieukerken EJV, Sohn J-C, Labandeira CC. 2015.** A revised checklist of Nepticulidae fossils (Lepidoptera) indicates an Early Cretaceous origin. *Zootaxa* **3963**:295–334.

**Friĉ A. 1882.** Fossile Arthropoden aus der Steinkohlen–und Kreideformation Böhmens. *Beiträge zur Paläontologie Österreich-Ungarns* **2**:1–7.

**Friĉ A. 1901.** Studien im Gebiete der Böhmischen Kreideformation. Palaentolologische Untersuchungen der einselnen Schichten. Die thierischen Reste der Perucer Schichten. *Archiv der naturwissenschaftlichen Landesdurch-forschung von Böhmen* **9**:163–181.

**Genise JF. 2017.** Insect Trace Fossils in Other Substrates than Paleosols I. Plant Remains. *Ichnoentomology: Insect Traces in Soils and Paleosols*. Cham: Springer International Publishing, 447–476.

**Givulescu R. 1984.** Pathological elements on fossil leaves from Chiuzbaia (galls, mines and other insect traces). *Dari de Seama ale Sedintelor, Intitutul de Geologie si Geofızica* **68**:123–133.

**Hering EM. 1930.** Eine Agromyziden-Mine aus dem Tertiär (Dipt. Agromyz.). *Deutsche Entomologische Zeitschrift, NF* **1931**:63–65.

**Hering EM. 1957.** *Bestimmungstabellen der Blattminen von Europa einschließlich des Mittelmeerbeckens und der Kanarischen Inseln*. 's-Gravenhage: Junk.

**Iannuzzi R, Labandeira CC. 2008.** The oldest record and early history of insect folivory. *Annals of the Entomological Society of America* **101**:79–94.

**Jakubowskaja TA.** **1955.** Sarmatikaja Flora Moldavskoja SSR. *Acta Inst. Bot. nom. V. L. Komarovii. Acad. Sci. USSR,* Ser. 1, Fasc. **11**:64–255. (in Russian)

**Kernbach K. 1967.** Über die bisher im Pliozän von Willershausen gefundenen Schmetterlings- und Raupenreste. *Bericht der Naturhistorischen Gesellschaft Hannover* **111**:103–108.

**Kinzelbach RK. 1970.** Eine Gangmine aus dem eozänen Ölschiefer von Messel (Insecta: ?Lepidoptera). *Paläontologische Zeitschrift* **44**:93–96.

**Krassilov VA, Rasnitsyn AP. 2008.** Plant-Arthropod Interactions in the Early Angiosperm History - Evidence from the Cretaceous of Israel. Sofia, Moscow, Leiden, Boston: Pensoft Publisher & BRILL.

**Kustatscher E, Franz M, Heunisch C, Reich M, Wappler T. 2014.** Floodplain habitats of braided river systems: depositional environment, flora and fauna of the Solling Formation (Buntsandstein, Lower Triassic) from Bremke and Fürstenberg (Germany). *Palaeobiodiversity and Palaeoenvironments* **94**:237–270.

**Labandeira CC. 2002.** Paleobiology of middle Eocene plant-insect associations from the Pacific Northwest: A preliminary report. *Rocky Mountain Geology* **37**:31–59.

**Labandeira CC. 2006.** The four phases of plant-arthropod associations in deep time. *Geologica Acta* **4**:409–438.

**Labandeira CC. 2013.** Deep-time patterns of tissue consumption by terrestrial arthropod herbivores. *Naturwissenschaften* **100**:355–364.

**Labandeira CC, Wilf P, Johnson KR, Marsh F. 2007.** *Guide to insects (and other) damage types on compressed plant fossils. Version 3.0*. Washington, D.C.: Smithsonian Institution.

**Lesquereux L. 1892.** The flora of the Dakota Group. *US Geological Survey Monograph* **17**:1–400.

**Mädler AK. 1936.** Eine Blattgalle an einem vorweltlichen Pappel-Blatt. *Natur und Volk* **66**:271–274.

**Peñalver E. 2002.** *Los insectos dípteros del Mioceno del Este de la Península Ibérica: Rubielos de Mora, Ribesalbes y Bicorp. Tafonomía y sistemática*. València: Universitat de València.

**Potonié H. 1893.** Die Flora des Rotliegendes von Thüringen. *Abhandlungen der Königlichen Preussischen Geologischen Landesanstalt*, NF **9**:1–298.

**Potonié R. 1921.** Mitteilungen über mazerierte kohlige Pflanzenfossilien. *Zeitschrift für Botanik* **13**:79–88.

**Robledo JM, Sarzetti LC, Anzótegui LM. 2016.** New records and ichnospecies of linear leaf mines from the late Miocene-Pliocene from Argentina and the establishment of leaf-mining ichnotaxobases. *Rivista Italiana di Paleontologia e Stratigrafia* **122**:73–88.

**Sarjeant WAS. 1979.** Code for trace fossil nomenclature. *Palaeogeography, Palaeoclimatology, Palaeoecology* **28**:147–167.

**Sarjeant WAS, Kennedy WJ. 1973.** Proposal of a Code for the nomenclature of trace fossils. *Canadian Journal of Earth Sciences* **10**:460–475.

**Sarzetti LC, Labandeira CC, Genise JF. 2008.** A leafcutter bee trace fossil from the Middle Eocene of Patagonia, Argentina and a review of megachilid (Hymenoptera) ichnology. *Palaeontology* **51**:933–941.

**Sarzetti LC, Labandeira CC, Muzón J, Wilf P, Cúneo NR, Johnson KR, Genise JF. 2009.** Odonatan Endophytic Oviposition from the Eocene of Patagonia: The Ichnogenus Paleoovoidus and Implications for Behavioral Stasis. *Journal of Paleontology* **83**:431–447.

**Simberloff D, Dayan T. 1991.** The guild concept and the structure of ecological communities. *Annual Review of Ecology and Systematics* **22**:115–143.

**Sohn J-C, Labandeira CC, Davis D, Mitter C. 2012.** An annotated catalog of fossil and subfossil Lepidoptera (Insecta: Holometabola) of the world. *Zootaxa* **3286**:1–132.

**Steinbach G. 1967.** Zur Hymenopterenfauna des Pliozäns von Willershausen/Westharz. *Bericht der Naturhistorischen Gesellschaft Hannover* **111**:95–102.

**Straus A. 1962.** Die Tongrube zu Willershausen. *Heimat-Kalender des Kreises Osterode und des Südwestrandes des Harzes* **1962**:65–68.

**Straus A. 1977.** Gallen, Minen und andere Fraßspuren im Pliozän von Willershausen am Harz. *Verhandlungen des Botanischen Vereins der Provinz Brandenburg* **113**:41–80.

**van Amerom HWJ. 1966.** *Phagophytichnus ekowskii* nov. ichnogen. & nov. ichnosp., eine Missbildung infolge von Insektenfrass, aus dem spanischen Stepanien (Provinz Leon). *Leidse Geologische Mededelingen* **38**:181-184.

**van Amerom HWJ,** **Boersma WJ. 1971.** A new find the ichnofossil *Phagophytychnus ekoswkii*. *Geologie en Mijnbouw* **50**: 667–670.

**von Heyden C. 1862.** Gliedertiere aus der Braunkohle des Niederrhein´s, der Wetterau und der Rhön. *Palaeontographica* **10**:62–82.

**von Kéler S. 1963.** *Entomologische Wörterbuch*. Berlin (Ost): Deutsche Akademie der Landwirtschaftswissenschaften zu Berlin.

**Vasilenko DV. 2005.** Damages on Mesozoic plants from the Transbaikalian locality Chernovskie Kopi. *Paleontological Journal* **39**:628–633.

**Vasilenko DV. 2007.** Feeding damage on upper Permian plants from the Sukhona River. *Paleontological Journal* **41**:207–211.

**Vialov OC. 1975.** The fossil traces of nourishment of the insects. *Paleontological Collection* **12**:147–155.

**Wedmann S, Wappler T, Engel MS. 2009.** Direct and indirect fossil records of megachilid bees from the Paleogene of Central Europe (Hymenoptera: Megachilidae). *Naturwissenschaften* **96**:703–712.

**Winkler IS, Labandeira CC, Wappler T, Wilf P. 2010.** Distinguishing fossil Agromyzidae (Diptera) leaf mines in the fossil record: new taxa from the Paleogene of North America and Germany and their evolutionary implications. *Journal of Paleontology* **84**:935–954.
