# Supplementary figures and images for: Plant-insect interactions patterns in three European paleoforests of the late-Neogene—early-Quaternary
Source: PeerJ. 2018 Jun 20;6:e5075. doi: 10.7717/peerj.5075 (PMC6015487; doi:10.7717/peerj.5075)

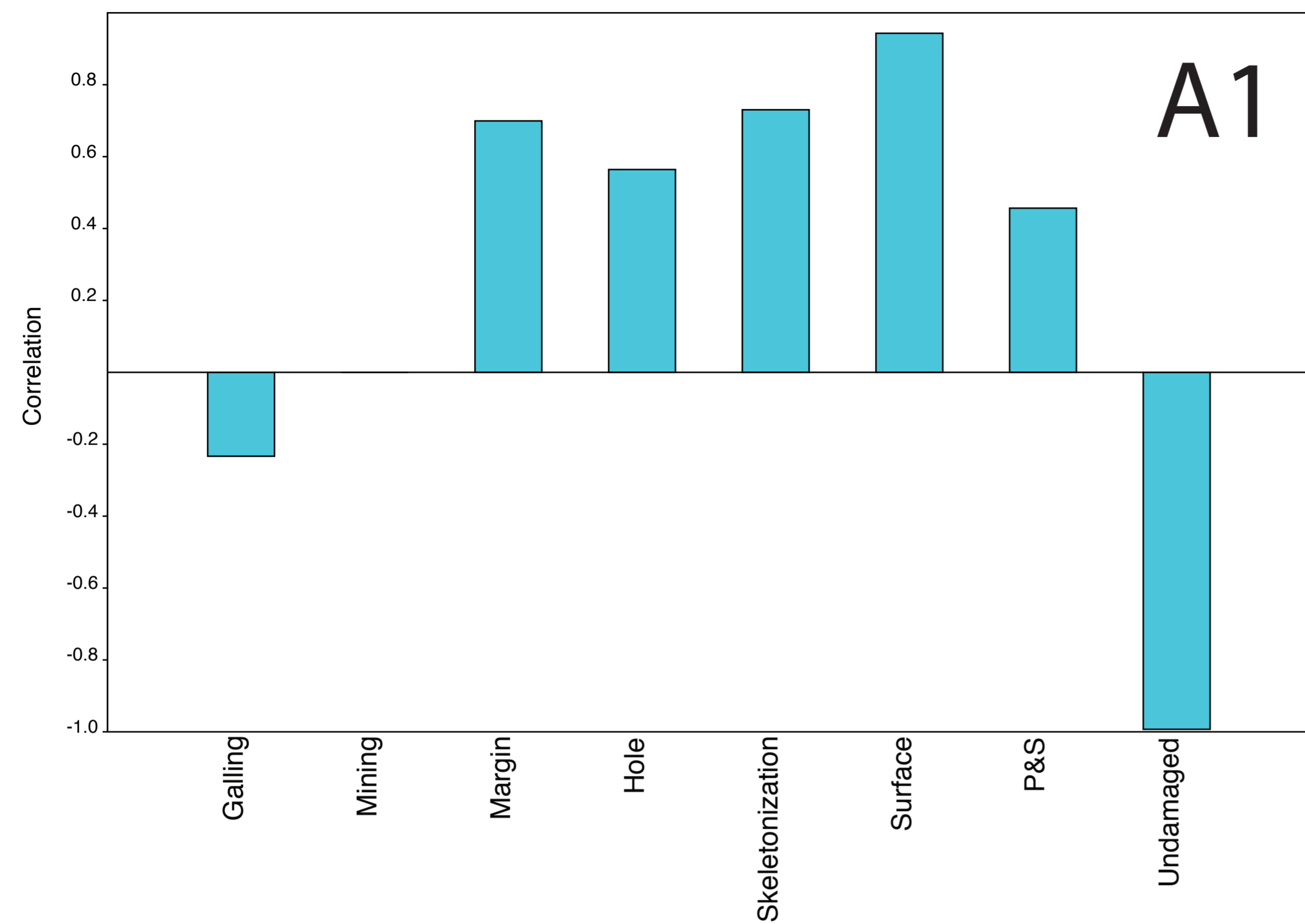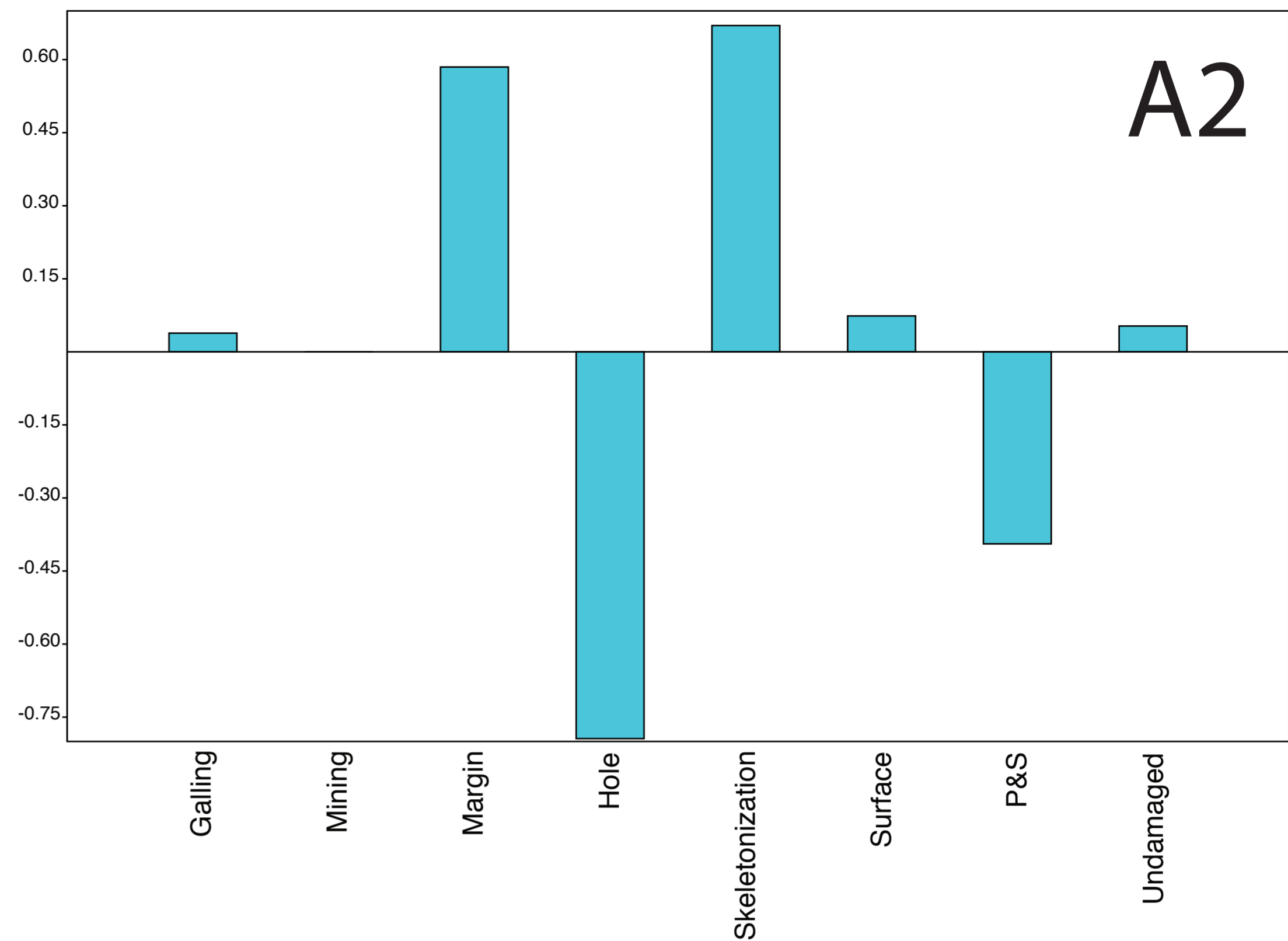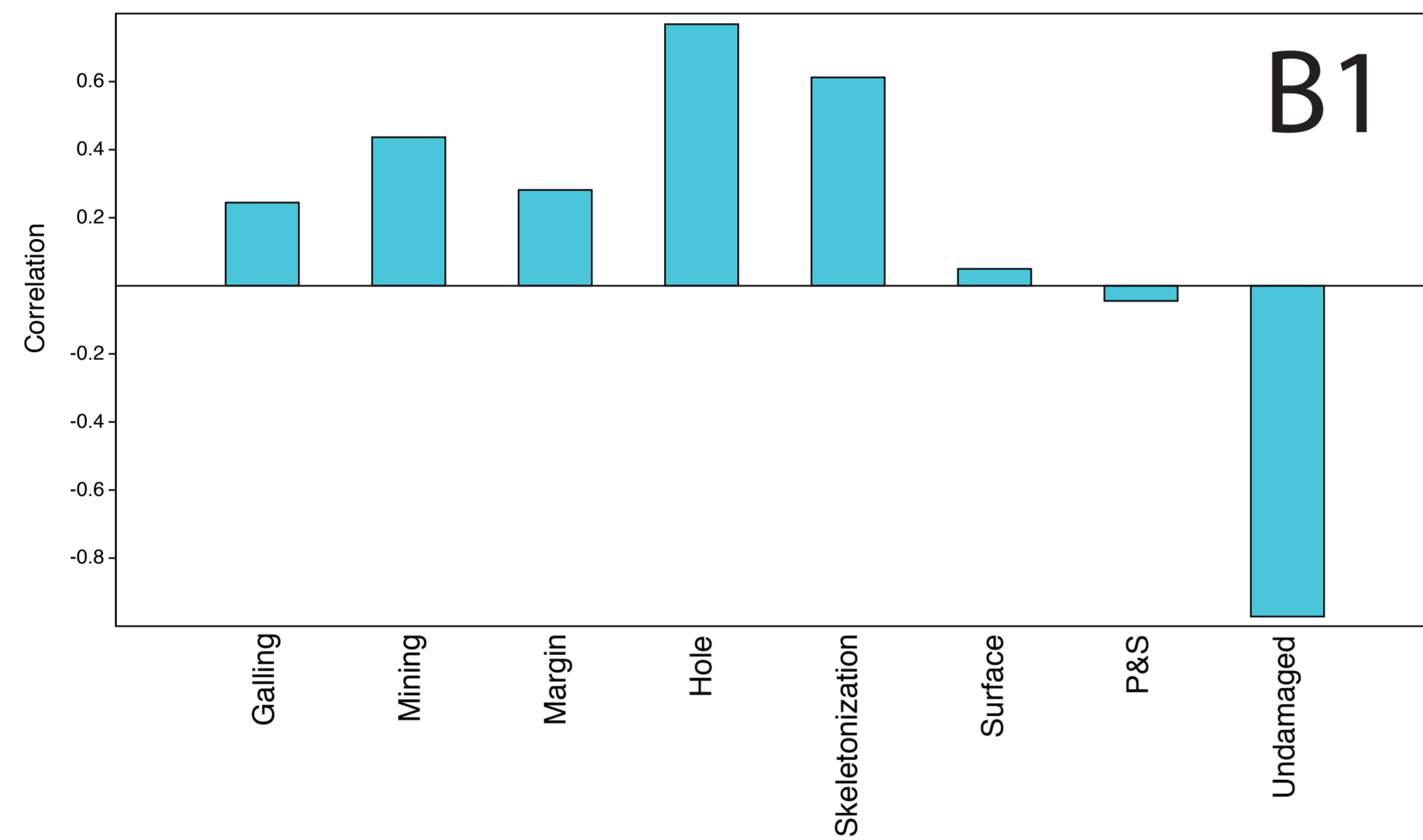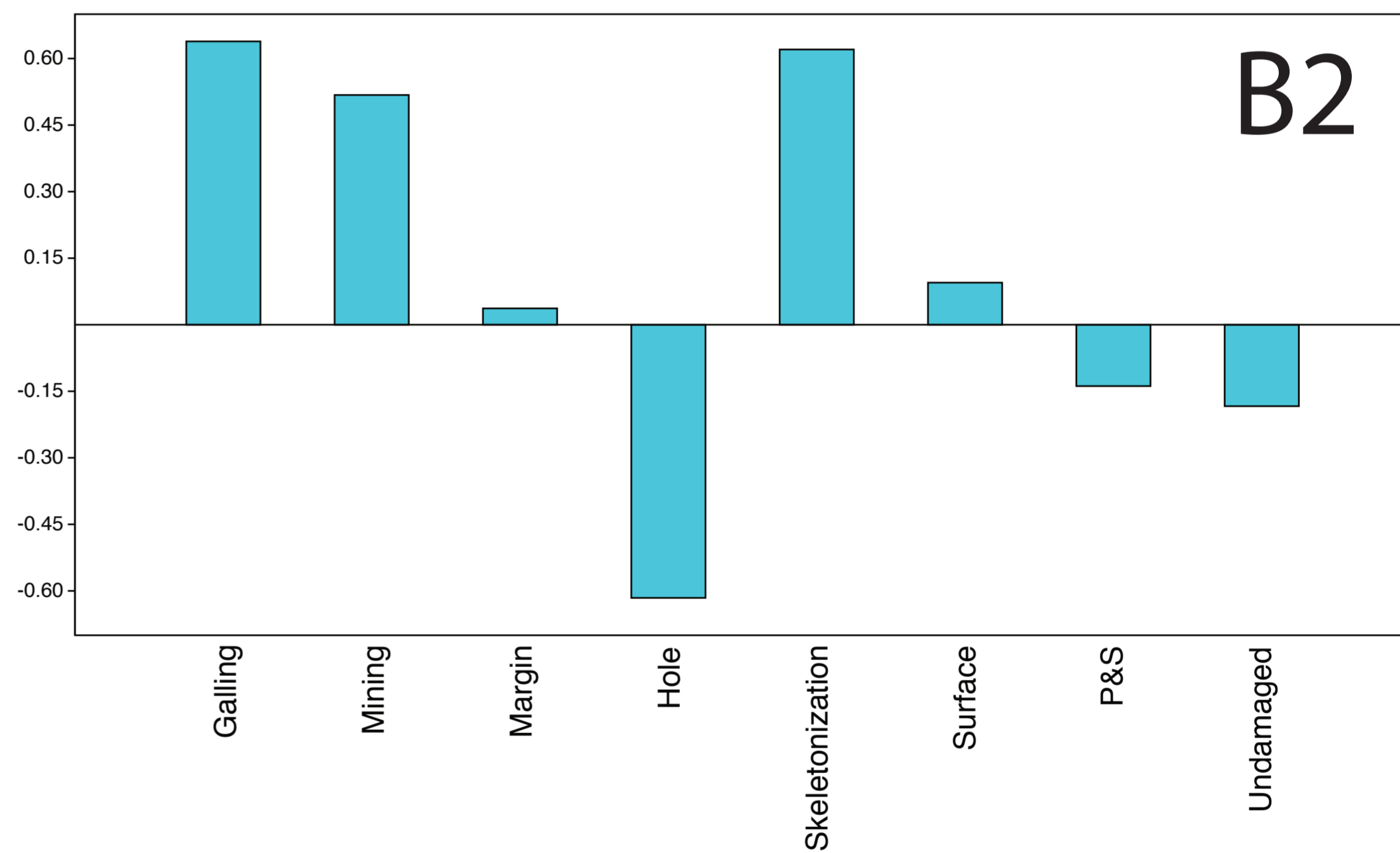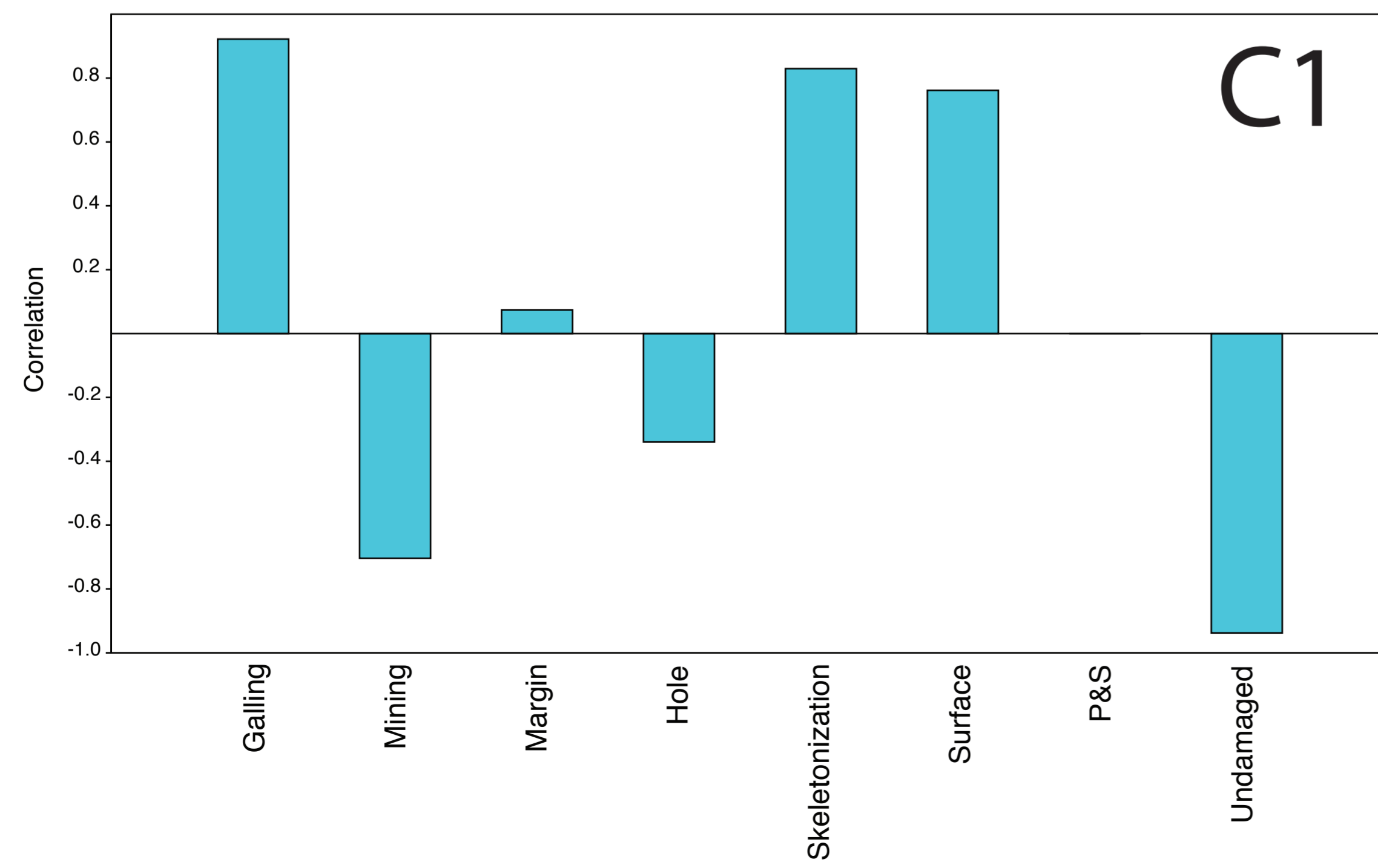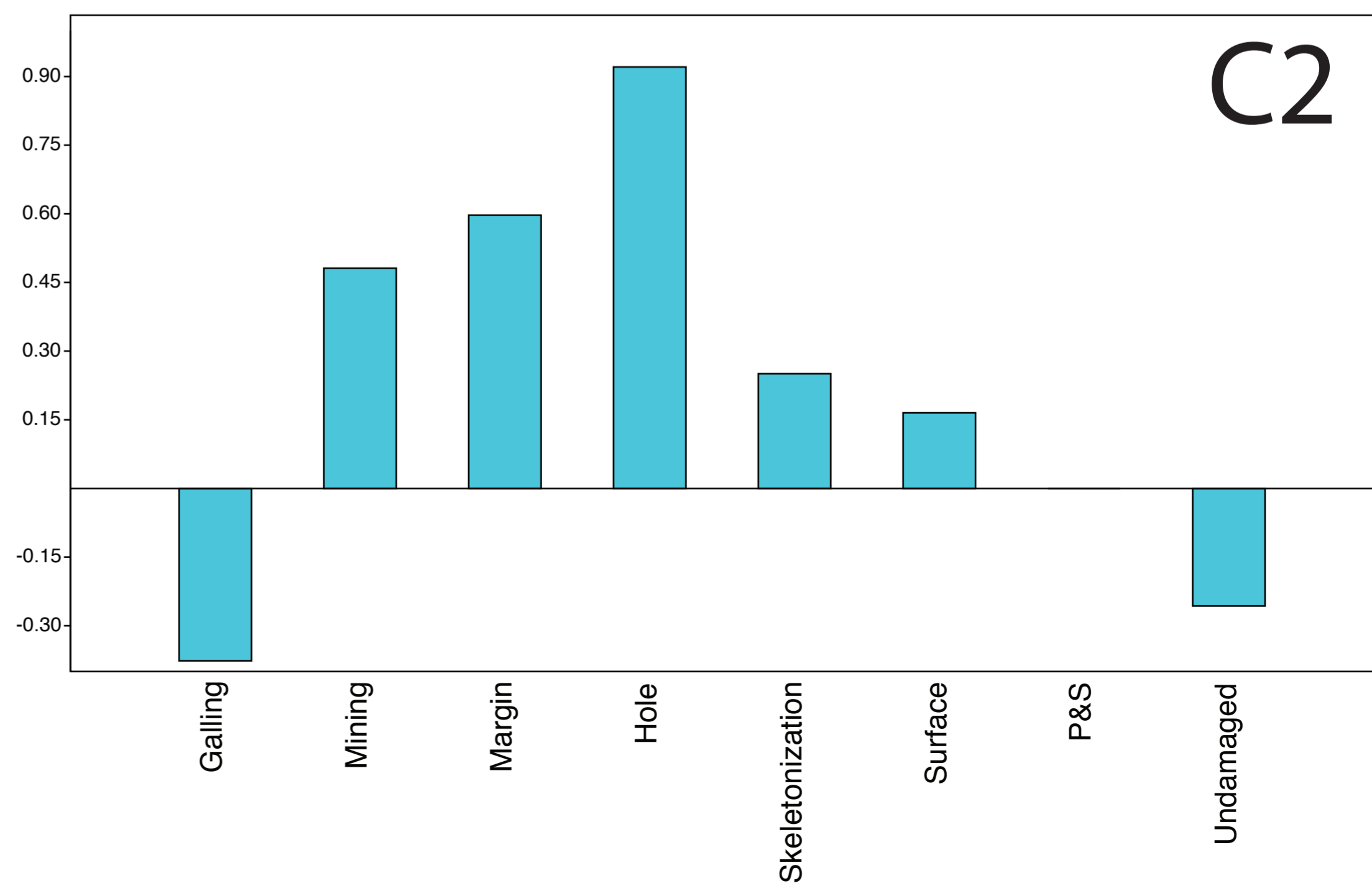

Supplement: Supplemental Information 4 [file peerj-06-5075-s004.pdf]
